# Supplementary material for: Muscle Oxygen Desaturation and Re-Saturation Capacity Limits in Repeated Sprint Ability Performance in Women Soccer Players: A New Physiological Interpretation
Source: Int J Environ Res Public Health. 2021 Mar 27;18(7):3484. doi: 10.3390/ijerph18073484 (PMC8037739; doi:10.3390/ijerph18073484)
Supplement: Supplementary file 1 [file ijerph-18-03484-s001.pdf]

Table S1. Correlation between muscle oxygen desaturation and re-saturation variables with workload variables during repeated-sprint ability in women soccer players.

Variables of SmO_2	Worst Time	Best Time	Mean Time	Max Power	Min Power	Mean Power	Fatigue Index	Heart Rate (ppm)	%Heart Rate Max	Speed (Km/h)	% Individual Speed	Efficiency Index
SmO_2 desaturation	0.401	0.162	231	-0.262	−0.434	−0.302	0.186	168	0.026	−0.251	0.156	−0.241
SmO_2 re-saturation	0.505 *	0.195	364	−0.348	−0.576 *	−0.426	0.234	132	−0.076	−0.374	−0.311	−0.319
Muscle Oxygen desaturation $_{rate}$	0.745**	127	507*	−0.377	−0.654 *	−0.524 *	0.670 *	−0.044	−0.356	−0.479	−0.597 *	−0.324
Muscle Oxygen resaturation $_{rate}$	−0.779 **	−0.177	−568 *	0.409	0.675 *	0.560 *	−0.696 **	0.054	0.357	0.538 *	0.625 *	−0.373
∇%SmO2	0.558*	0.162	0523 *	−0.268	−0.443	−0.422	0.635 *	−298	−397	−0.479	−0.609 **	−0.165

Statistical analysis of Pearson correlation and interpreted as follows: trivial (0.00–0.09), small (0.10–0.29), moderate (0.30–0.49),* large (0.50–0.69), ** very large (0.70–0.89), *** nearly perfect (0.90–0.99), and perfect (1.00).
